# Clinical Utility of Medication-Based Risk Scores to Reduce Polypharmacy and Potentially Avoidable Healthcare Utilization

**DOI:** 10.3390/ph15060681

**Published:** 2022-05-28

**Authors:** Armando Silva-Almodóvar, Milap C. Nahata

**Affiliations:** 1 Institute of Therapeutic Innovations and Outcomes (ITIO), College of Pharmacy, Ohio State University, Columbus, OH 43210, USA; silvaalmodovar.1@osu.edu; 2 Tabula Rasa HealthCare, Tucson, AZ 85701, USA; 3 College of Medicine, Ohio State University, Columbus, OH 43210, USA

**Keywords:** medication-based risk score, deprescribing, polypharmacy, health outcomes

## Abstract

The management of multiple chronic health conditions often requires patients to be exposed to polypharmacy to improve their health and enhance their quality of life. However, exposure to polypharmacy has been associated with an increased risk for adverse effects, drug-drug interactions, inappropriate prescribing, medication nonadherence, increased healthcare utilization such as emergency department visits and hospitalizations, and costs. Medication-based risk scores have been utilized to identify patients who may benefit from deprescribing interventions and reduce rates of inappropriate prescribing. These risk scores may also be utilized to prompt targeted discussions between patients and providers regarding medications or medication classes contributing to an individual’s risk for harm, eventually leading to the deprescribing of the offending medication(s). This opinion will describe existing medication-based risk scores in the literature, their utility in identifying patients at risk for specific adverse events, and how they may be incorporated in healthcare settings to reduce rates of potentially inappropriate polypharmacy and avoidable healthcare utilization and costs.

## 1. Introduction

Patients with multiple chronic conditions may be exposed to several medications to improve health, enhance quality of life, and reduce healthcare utilization. The chronic exposure of multiple medications has been termed polypharmacy [1]. While the operational definition of polypharmacy may vary, from utilizing ≥2 to ≥11 medications [1], polypharmacy is associated with an increased risk of drug-drug interactions, drug-related problems, inappropriate prescribing, medication non-adherence, and increased healthcare utilization such as emergency department visits, hospitalizations, and medical costs [2]. 

Medication-based risk scores can be utilized by healthcare providers to identify patients who would benefit from deprescribing interventions to reduce rates of potentially inappropriate polypharmacy, adverse health outcomes, and avoidable healthcare utilization. The utilization of these tools is especially important due to the increasing proportions of adults with multiple chronic conditions, resulting in higher prevalence of polypharmacy [3]. The higher prevalence of polypharmacy has led to an increased risk for clinically relevant drug-drug and drug-disease interactions. This opinion will describe medication-based risk scores that can be used to identify patients at substantial risk for experiencing clinically significant adverse outcomes, propose their utility in identifying medication targets for deprescribing, and suggest how these tools can be incorporated into clinical practice or clinical decision support systems. 

## 2. Medication-Based Risk Scores

### 2.1. Medication Regimen Complexity Index (MRCI)

The MRCI is a risk assessment tool that quantifies the complexity of an individual’s medication regimen by taking into consideration the medication dosage form and route, dosing frequency, and unique directions provided to take certain medications [4]. This tool utilizes a continuous scale where higher scores suggest that an individual’s medication regimen is more complex [4]. A systematic review found higher MRCI to be associated with medication nonadherence, hospital readmission, and lower quality of life [5]. While some studies found patients with scores greater than a certain number to be associated with worsening health outcomes in select populations [5], there are no validated parameters suggesting which values signal a clearly increased risk for adverse events. Future research is warranted to determine specific scores to be utilized as surrogate markers to identify patients likely to benefit most from deprescribing interventions. It is important to note that while the MRCI score is typically calculated manually due to the consideration of unique medication directions, a study by McDonald et al. [6] described how to automate the calculation of MRCI within an electronic health record. 

### 2.2. Medication Complexity Score (MCS)

The development of the MRCI allowed clinicians and researchers to quantify the complexity of an individual’s medication regimen. However, since part of the score requires the use of unique prescribing details [5], the score cannot be calculated using only prescription claims data. This is a potential weakness, as prescription claims data may be better suited to capture prescribing patterns across multiple health systems and prescribers [7]. The MCS was developed and modeled against the MRCI to demonstrate its comparability as a tool in identifying medication burden and risk for greater healthcare utilization with the use of just prescription claims [7]. The MCS utilizes a prescription claim’s national drug code to infer the drug’s dosage form and route; the days’ supply and number of units are utilized to infer a dosing frequency. The final component of the MRCI, being unique medication instructions, was not adapted to the MCS. As with MRCI, future research identifying specific cut-off values of the MCS score signifying which patients would benefit most from deprescribing interventions would be beneficial.

### 2.3. Medication Fall Risk Score (MFRS)

Just under 1,000,000 patients fall within healthcare facilities each year in the United States (US) [8]. Reducing the prevalence of falls within facilities is necessary to ensure positive patient outcomes while reducing avoidable costs incurred by patients and healthcare facilities. Specific medications are important risk factors to consider when evaluating a patient’s risk of falling due to their mechanisms of action being associated with greater risk of dizziness, sedation, impaired cognition, and changes in blood pressure [8]. The MFRS incorporates the prescribing of specific medication classes (e.g., antipsychotics, benzodiazepines, antiarrhythmics, antidepressants) to determine the associated risk of incurring a fall. Medications are weighted and summed to determine an individual’s risk of falling. Patients with a score ≥6 are at “a higher risk for falling” and are further evaluated using medication fall risk evaluation tools [9]. In addition to the implementation of further medication reviews and local clinical interventions specific to healthcare settings, patients identified as being at an increased risk of falls may benefit from deprescribing interventions to reduce their risk of falls.

### 2.4. Medication-Based Index of Physical Function (MedIP)

Another tool used to measure fall risk is the MedIP. This tool was developed to overcome biases from tools that focus on specific medication classes [10]. The MedIP utilizes medications included in the Side Effect Resource (SIDER) dataset, a resource that obtains side effect related information of medications from public sources such as package inserts [11]. The MedIP calculates a sum utilizing elementary matrix operations that take into consideration the drugs being used by a patient, their risk of side effects and the contribution of side effects to fall risk based on data found within SIDER. While higher scores indicate a greater risk of falling, the validation study estimated a score of 2764 to have optimal sensitivity and specificity in predicting fall risk [10].

### 2.5. Drug Burden Index (DBI)

The use of medications with sedative and anticholinergic properties has been associated with adverse events among older adults including cognitive impairment and falls [12]. The DBI score takes into consideration the dose and exposure of medications with anticholinergic and/or sedative effects on patients [13]. This score utilizes an algorithm that sums the sedative and anticholinergic weighted burden of each medication to generate a score. Each medication weight is calculated by dividing the daily dose with the sum of the minimum recommended daily dose and the daily dose [12]. Higher scores suggest a higher anticholinergic and sedative burden; studies have generally evaluated healthcare utilization risk comparing patients with scores greater than 0 or 1 versus lower scores [12].

While the findings of studies assessing the relationship between DBI and healthcare utilizations and outcomes such as falls have varied, higher DBI has been consistently shown to be associated with frailty, poorer quality of life, and physical impairment [12]. However, it is important to note the DBI score has several limitations: medications with relevant anticholinergic and sedative effects are considered equivalent (there is no adjustment for medications having stronger or weaker sedative or anticholinergic effects), and there is presently not an updated consensus document listing medications to consider for the determination of DBI. It is also important to note that the DBI was developed utilizing the minimum daily dose as indicated by the United States Food and Drug Administration. Given that minimum daily doses may vary among countries and with indications for use, a DBI algorithm was developed utilizing a defined daily dose published by the World Health Organization (WHO) to facilitate use of this algorithm across different countries [14].

### 2.6. Anticholinergic Burden Medication Based Risk Scores

Medications contributing to anticholinergic burden is a field of significant interest due to widely used drug classes with anticholinergic properties being associated with significant healthcare utilization and poor health outcomes. This has led to the publication of numerous scales, lists, and risk scores that quantify an individual’s anticholinergic burden to estimate their risk of adverse outcomes. Furthermore, given the substantial interest and breadth of research examining anticholinergic burden, several systematic reviews have been published examining the utility of these scales to predict poor health outcomes such as adverse events, falls, mortality, delirium, and poor quality of life [15,16,17,18,19,20,21,22,23,24]. 

Scores, scales, and lists available to measure anticholinergic burden or identify exposure to anticholinergic medications include the anticholinergic drug scale [25] (ADS), anticholinergic burden classification [26] (ABC), anticholinergic effect on cognition [27] (AEC), anticholinergic risk scale [28] (ARS), anticholinergic cognitive burden scale [29] (ACBS), anticholinergic activity scale [30] (AAS), anticholinergic loading scale [31] (ALS), Korean anticholinergic burden scale [32], German anticholinergic burden score [33], Brazilian anticholinergic activity scale [34], Cancelli’s ACH burden scale [35], Aizenberg’s Anticholinergic Burden Scale [36], Duran’s Anticholinergic Burden Scale [17], Salahudeen’s Anticholinergic Burden Scale [15], Summers drug risk number [37], Whalleys Anticholinergic Burden Scale [38], Chew’s list of anticholinergic drugs [39], Clinician-Rated Anticholinergic Score [40], Minzenberg’s Clinical Index and Pharmacological Index [41], anticholinergic impregnation scale [42], a modified ARS [43], modified ACB [44], deliriogenic risk scale [45], and the anticholinergic toxicity scale [46].

These tools provide prescribers with an understanding of a patient’s risks for specific adverse outcomes based on their cumulative exposure to medications with anticholinergic activity. However, despite the large number of tools available to highlight anticholinergic burden, there are several details to consider. Significant variability exists between tools, with some taking into consideration the dose of medications, and several tools identifying <30 medications and others considering >500 medications with expected anticholinergic activity [22]. Furthermore, while agreement on the level of anticholinergic activity may vary between tools, it is unclear which specific tool is best. Providers may benefit from using systematic reviews that have compiled lists of medications across multiple scales to comprehensively define which medications had low, moderate, and high anticholinergic activity [15,17,22]. Published systematic reviews of these tools have provided greater detail of the strengths and weaknesses of these tools and their associations with poor health outcomes [15,16,17,18,19,20,21,22,23,24].

### 2.7. Sedative Load Model (SLM)

The SLM was designed to characterize an individual’s exposure to medications with sedative properties and to quantify their risk of impaired mobility [47,48]. This risk is calculated by summing the weights of medications contributing to an individual’s sedative burden. Medications considered a primary sedative included a score of 2, while medications with a major side effect or with ingredients considered potentially sedating were given a score of 1 [47]. A higher sedative load is associated with impaired mobility [47]. While additional research is needed in diverse older adult populations, higher sedative load has been associated with incident delirium and falls among patients with Alzheimer’s disease [49].

### 2.8. Sloan Sedative Risk Score

Sloan et al., modified the SLM to construct a sedative load risk score that incorporated the dose of a medication as well [50]. This risk score applies weights differently, with psychotropic medications intended to cause sedation receiving a weight of 6, while medications with sedation as a common side effect were given a weight of 3, and medications at a low-risk of sedation side effect were given a weight of 1 [50]. The dose of each medication is divided by the mean effective dose, which is then multiplied by the assigned weight and summed for a final score [50]. While the utilization of this model can describe the sedative risk of a population, with higher scores implying greater risk, it is unclear if there is a specific score associated with the significantly increased risk of falling or other adverse outcomes related to sedation.

### 2.9. Central Nervous System (CNS) Medication Burden

Another measure that was developed to quantify an individual’s medication related risk for falls included the CNS medication burden [51]. This risk score is calculated by summing the daily dose of each CNS medication with each divided by the minimum effective geriatric daily dose [51]. Individuals with scores ≥3 are considered at greater risk of experiencing serious falls [51,52]. Future research would be beneficial to assess if medication-based interventions that reduce an individual’s medication exposure subsequently reduce their risk of falls.

### 2.10. Medication Appropriateness Index (MAI)

A challenge in deprescribing medications is the identification of prescriptions that are appropriate and inappropriate. While the previously noted risk scores identified medications or medication classes that were potentially inappropriate for an individual, the MAI is a scoring system that determines if a medication is inappropriate and should be targeted for modification or deprescribing. The original tool utilizes 10 questions where a clinician assesses if a drug is indicated, effective, appropriately dosed, given with appropriate instructions, practical to use, prescribed with appropriate length of therapy, relatively affordable compared to similar drugs, and does not have any clinically significant drug-drug or drug-disease interactions [53]. A clinician must determine if a drug is appropriate (score = 1), inappropriate (score = 3), or marginally appropriate (score = 2), with each drug having a maximum score of 18; higher scores would suggest that a drug may be inappropriate. The sum of the scores is used to determine an individual’s exposure to inappropriate medications [53]. The tool has been validated in multiple settings comparing responses among clinicians to ensure consistency in its application across various practitioners [54]. Additionally, the MAI has been modified to a three-item survey and adapted and validated in various settings [55].

Despite the advantages of utilizing an individual clinician’s knowledge to determine the inappropriateness of a medication or medication regimen, there are important limitations of MAI. Agreement between clinicians on the determination of the inappropriateness of a medication may improve after discussion, suggesting that interpretation of inappropriateness based on patient specific factors and identification of clinically relevant drug-drug or drug-disease interactions can differ based on a provider’s experience and background [56]. Furthermore, assessing for the appropriateness of medications based on price or practicality may benefit from patient input. Finally, the tool may take up to 10 min to properly evaluate one drug, therefore it may not be practical to use for patients on multiple medications in most settings [53].

### 2.11. MedWise Risk Score

Previous medication-based risk scores may be limited in their utility given that they are used to track one or two specific risk factors in a patient’s medication regimen, such as fall risk, anticholinergic burden, sedative load, complexity, or appropriateness. In contrast, the MedWise Risk Score measures an individual’s risk for adverse events based on specific risk factors including sedative load, anticholinergic burden, competitive CYP450 drug interaction burden, risk of QT prolongation, and risk for adverse events utilizing the FDA Adverse Event Reporting System [57]. This score exists on a continuous scale with higher scores being associated with adverse events, healthcare related costs, emergency room visits, falls, and mortality [57,58,59]. Furthermore, use of the MedWise Risk Score as part of medication risk mitigation services may significantly reduce healthcare costs related to emergency room visits, hospital admissions, and skilled nursing visits for organizations providing services for older adults that require nursing facility level care [60].

### 2.12. Medication Intensity Scale (MIS)

An important goal of care among patients with asthma is to improve quality of life and reduce healthcare utilization such as emergency room visits and hospitalizations. In 2013, asthma was associated with approximately $50 billion in medical costs in the United States [61]. The MIS is one means of identifying patients with suboptimal control of their asthma to target resources and reduce the prevalence of potentially avoidable healthcare costs. The MIS is a four-point scale (0–3) that ascribes a point to a patient for having 5–13 beta-agonists canisters, >13 beta-agonist canisters, and having greater than two dispensations of oral corticosteroids within a year [62]. Validation of this tool demonstrated that higher scores were associated with greater emergency department utilization [62]. The validation study of this tool recommends that the cut-off chosen for intervention be determined by the cost of the intervention; when it was given using lower cut-offs it substantially increased the false positive rate of patients with asthma at risk for utilizing the emergency department [62]. The automated monitoring of prescription claims could identify patients with asthma at greatest need of intervention to improve asthma control and reduce potentially avoidable healthcare utilization.

## 3. Implementation within Healthcare Systems

Medication-based risk scores are a set of tools that can be utilized at the healthcare system level and the individual prescriber level to identify patients at significant risk for experiencing specific adverse events or potentially avoidable healthcare utilization. While risk scores have been traditionally utilized as tools for risk adjustment and prediction of healthcare utilization [63], medication-based risk scores can be utilized by providers to identify patients at greater risk for specific adverse events and reduce potentially inappropriate polypharmacy. The use of medication-based risk scores may prompt discussions between patients and providers regarding medications contributing to an individual’s risk for harm, eventually leading to the deprescribing of the offending medication. Furthermore, given their dependence on only medication-related information, these risk scores can be operationalized in settings where prescribing occurs, or prescription claims data is accessible. However, prior to the incorporation of medication-based risk scores into healthcare services, there are important details to consider.

The use of medication-based risk scores should be used with resources that facilitate deprescribing interventions. While most medication-based risk scores are validated to detect an increased risk of specific adverse events or healthcare utilization, they have not been studied extensively in their efficiency in identifying medications that should be deprescribed nor have they been compared to each other in this respect. It is important to note that medication-based risk scores should not solely be used to determine the appropriateness or inappropriateness of a medication. They should be used to complement a comprehensive evaluation of an individual’s medications. These tools are used to identify potential targets for deprescribing and enhance the quality of information contributing to the risk-benefit assessment of an individual’s prescriptions. Within primary care and outpatient settings, these risk scores can be used to identify patients who may benefit from deprescribing before a potentially avoidable event occurs, while hospital or emergency room settings can utilize these risk scores to compliment the identification of adverse events related to medication use. Hospital settings may benefit more from using risk scores that incorporate physiological data to identify emergent events that require prompt intervention.

Providers need to evaluate which risk scores are most useful in identifying patients at greatest risk for specific harms or healthcare utilization within their specific healthcare settings. Presently, each risk score describes the risk for exposure to certain medications and medication classes in relation to specific adverse outcomes. It is also important to realize that risk scores alone do not overcome barriers to deprescribing such as managing interprofessional relationships, increasing provider workload, the reluctance to discontinue chronic medications, or differences in knowledge between providers [64]. Some of these barriers can be overcome with the use of deprescribing algorithms and guidelines that provide steps and rationale to safely and efficiently deprescribe certain medications [65].

Primary care and outpatient healthcare settings may want to utilize medication management programs or pharmacovigilance services to monitor the use and prescribing of medications without additional work and burden for prescribers. Alternatively, insurance plan providers can utilize prescription claims data to identify patients at greater risk of adverse events based on their prescribing data. Medication management programs can be utilized to communicate with the providers of these patients to prompt review of their medications to consider deprescribing interventions. The implementation of medication reviews at the insurance claims level can overcome challenges associated with patient fragmentation of data across various healthcare and dispensing settings. Additionally, having personnel specialized in the deprescribing of medications can ensure that medication-based risk scores and deprescribing tools are used optimally and efficiently among patient populations. Patients with higher risk for medication related adverse events may benefit from periodic medication reviews where medications with the lowest benefit to harm ratio are targeted for deprescribing [66].

## 4. Conclusions

Medication-based risk scores are useful in identifying patients potentially at risk for suboptimal health outcomes and avoidable healthcare utilization and adverse events. To ensure that healthcare settings are able to efficiently reduce harms associated with exposure to the medications, combining these tools with deprescribing algorithms and guidelines may facilitate the discontinuation of medications that provide the least benefit and most harm to patients. The utilization of pharmacovigilance and medication management programs may be implemented within healthcare settings to identify opportunities for deprescribing and reduce potentially avoidable healthcare utilization.

## Data Availability

Data is contained within the article.
